# Molecular dynamics and protein frustration analysis of human fused in Sarcoma protein variants in Amyotrophic Lateral Sclerosis type 6: An *In Silico* approach

**DOI:** 10.1371/journal.pone.0258061

**Published:** 2021-09-29

**Authors:** L. F. S. Bonet, J. P. Loureiro, G. R. C. Pereira, A. N. R. Da Silva, J. F. De Mesquita

**Affiliations:** Department of Genetics and Molecular Biology, Laboratory of Bioinformatics and Computational Biology, Federal University of the State of Rio de Janeiro, Rio de Janeiro, Brazil; Consejo Superior de Investigaciones Cientificas, SPAIN

## Abstract

Amyotrophic lateral sclerosis (ALS) is the most frequent adult-onset motor neuron disorder. The disease is characterized by degeneration of upper and lower motor neurons, leading to death usually within five years after the onset of symptoms. While most cases are sporadic, 5%-10% of cases can be associated with familial inheritance, including ALS type 6, which is associated with mutations in the Fused in Sarcoma (*FUS*) gene. This work aimed to evaluate how the most frequent ALS-related mutations in FUS, R521C, R521H, and P525L affect the protein structure and function. We used prediction algorithms to analyze the effects of the non-synonymous single nucleotide polymorphisms and performed evolutionary conservation analysis, protein frustration analysis, and molecular dynamics simulations. Most of the prediction algorithms classified the three mutations as deleterious. All three mutations were predicted to reduce protein stability, especially the mutation R521C, which was also predicted to increase chaperone binding tendency. The protein frustration analysis showed an increase in frustration in the interactions involving the mutated residue 521C. Evolutionary conservation analysis showed that residues 521 and 525 of human FUS are highly conserved sites. The molecular dynamics results indicate that protein stability could be compromised in all three mutations. They also affected the exposed surface area and protein compactness. The analyzed mutations also displayed high flexibility in most residues in all variants, most notably in the interaction site with the nuclear import protein of FUS.

## Introduction

Amyotrophic Lateral Sclerosis (ALS) is a neurodegenerative disease characterized by damage to upper and lower motor neurons, resulting in progressive muscle atrophy, paralysis, and usually death due to respiratory failure within 1 to 5 years after the onset of symptoms [1]. With an annual incidence of 2 per 100000 people worldwide, ALS is the most common adult-onset motor neuron disorder [2]. Despite the vast literature on the disease, its physiological basis is still poorly understood, being considered a multifactorial condition in about 90–95% of cases [3]. The remaining 5–10% can be associated with familial inheritance, termed familial ALS (fALS), with several known genes relating to ALS either causatively or as susceptibility factors [4].

Among these genes, the Fused in Sarcoma (*FUS*) is associated with approximately 5% of fALS cases [5]. FUS is a DNA- and RNA-binding protein associated with splicing regulation, stress granule formation, mRNA transport, and DNA repair [6]. FUS is a nuclear protein composed of 526 amino acid residues and seven domains [7]. Notably, most of the *FUS* mutations associated with ALS development occur in the protein’s C-terminal region [8], specifically in its nuclear localization signal (NLS) domain. Among these non-synonymous single nucleotide polymorphisms (nsSNP), variant R521C is the most frequent mutation in ALS type-6 patients [5,9,10]. Variants R521H and P525L follow closely as second and third most common [6] mutations in ALS type 6 cases. Additionally, P525L has been associated with a more aggressive disease phenotype [6].

There are very few treatment options for ALS patients, with merely two approved drugs, both of which increase patient lifetime by 3–5 months [11]. The lack of knowledge regarding the pathogenesis of the disease contributes to the absence of effective treatments [12]. Nevertheless, advances in computational technologies and applied bioinformatics have allowed scientists a new perspective on the molecular basis of diseases, which is an essential piece in rational drug design and precision medicine [13]. Here, we performed molecular dynamics to better understand how ALS-related mutations in FUS behave inside a simulated cellular environment and how this behavior can be associated with fALS development. Furthermore, we used several computational algorithms to predict the effects of mutations R521C, R521H, and P525L and analyze the evolutionary conservation of amino acids in the human FUS protein.

## Materials and methods

We used the methodology previously described by our group [13–15] to analyze the effects that mutations R521C, R521H, and P525L have over the human FUS protein.

### Ethics statement

This research does not involve human participants or human tissues. All data used as a starting point for our study were obtained from freely available databases, such as Protein Data Bank and UniProt. No participant consent or ethics committee approval is needed in this case.

### Sequence and structure retrieval

The wild-type sequence of the human FUS protein, as well as a list of its naturally occurring variants, were obtained from the UNIPROT [16] database [ID: P35637]. The structural models for the human FUS protein were obtained from the Protein Data Bank [17]. As there is no model for the complete protein structure, two fragments containing the nuclear localization signal of FUS wild-type [ID: 4FDD] [18] and variant P525L [ID: 7CYL] [19] were selected for computational analysis after meticulous research showed that molecular dynamics simulations (MD) involving protein fragments were viable [20,21]. Both fragments ranged from position 507 to 526 of the human FUS protein.

### nsSNP analysis

Ten algorithms were used to predict whether the variants could cause deleterious functional alterations on the protein: PredictSNP [22], MAPP [23], PhD-SNP [24], Polyphen-1 [25], Polyphen-2 [26], SIFT [27], SNAP [28], SNPs&GO [29], PMut [30] and PROVEAN [31]. Additional factors, such as aggregation tendency (TANGO), amyloid propensity (WALTZ), chaperone binding tendency (LIMBO), and protein stability were analyzed using the SNPeffect [32] and FoldX [33] algorithms.

### Protein frustration analysis

To investigate if the studied mutations caused changes in protein frustration levels, the wild-type structure and the variant structures were analyzed in the Protein Frustratometer 2 server [34]. The variant structures (R521C, R521H, and P525L) were generated using the model obtained from the PDB and the *in silico* mutagenesis extension of the Visual Molecular Dynamics software [35], version 1.9.3.

### Evolutionary conservation analysis

The CONSURF algorithm was used to evaluate the evolutionary conservation of amino acid residues in the FUS protein [36]. UNIPROT was selected as the database, and the homolog search algorithm used was HMMER, in 1 iteration and an E-value cutoff of 0.0001. Maximum and minimum identities for homologs were set as 95% and 35%, respectively The alignment method used to build the Multiple Sequence Alignment was MAFFT-L-INS-i. The calculation method used was the Bayesian method, and the evolutionary substitution model was set to default.

### Molecular dynamics

The wild-type and variant models of the FUS protein were submitted to molecular dynamics using the Linux GROMACS [37] package, version 5.0.7. Using the Amber99SB-ILDN [38] force field, the protein fragment was solvated in TIP3P [39] water molecules inside a triclinic box with dimensions of 56Å, 34Å, and 31Å. The dynamics were neutralized by the addition of Na^+^ and Cl^-^ ions. The systems underwent energy minimization using the steepest descent method. After minimization, NVT (constant number of particles, volume, and temperature) and NPT (constant number of particles, pressure, and temperature) ensembles were performed, both at a pressure of 1atm and temperature of 300K for 100ps. The Linear Constraint Solver (LINCS) algorithm [40] was utilized to contain covalent bonds, while the electrostatic interactions were computed using the Particle Mesh Ewald (PME) method [41]. Production simulations were performed at 300K and 1atm for 300ns for the wild-type FUS and variants.

Three independent experiments were performed for the wild-type protein and its variants. The resulting trajectories were analyzed using the following GROMACS distribution programs: *gmx rms*, *gmx rmsf*, *gmx gyrate* and *gmx sasa*, which generated parameter values for Root-mean-Square Deviation (RMSD), Root-mean-Square Fluctuation (RMSF), radius of gyration (Rg), and solvent-accessible surface area (SASA). The ggplot2 package from the R software was used to generate the MD simulation graphs and calculate the mean values for the wild-type FUS and variants among the triplicates.

## Results and discussion

### nsSNP analysis

Most of the algorithms accurately predicted the mutations R521C, R521H, and P525L to be deleterious, as shown in Table 1. PredictSNP, MAPP, Polyphen-1, Polyphen-2, SIFT, SNAP, and Pmut were the most accurate predictors, classifying all three mutations as deleterious, while PHD-SNP and SNPs&GO classified all three as neutral. Interestingly, PROVEAN classified variants R521C and P525L as deleterious but R521H as neutral.

**Table 1:** Functional effect prediction of FUS mutations by ten nsSNP prediction algorithms.

	Protein Variants		
Prediction algorithms	R521C	R521H	P525L
PredictSNP	Deleterious [72%]^1^	Deleterious [72%]^1^	Deleterious [65%]^1^
MAPP	Deleterious [77%]^1^	Deleterious [56%]^1^	Deleterious [86%]^1^
PHD-SNP	Neutral [78%]^1^	Neutral [72%]^1^	Neutral [78%]^1^
Polyphen-1	Deleterious [74%]^1^	Deleterious [59%]^1^	Deleterious [59%]^1^
Polyphen-2	Deleterious [55%]^1^	Deleterious [55%]^1^	Deleterious [56%]^1^
SIFT	Deleterious [79%]^1^	Deleterious [79%]^1^	Deleterious [79%]^1^
SNAP	Deleterious [89%]^1^	Deleterious [81%]^1^	Deleterious [56%]^1^
SNPs&GO	Neutral [0.114]^2^	Neutral [0.072]^2^	Neutral [0.039]^2^
PMut	Deleterious [87%]^3^	Deleterious [86%]^3^	Deleterious [91%]^3^
PROVEAN	Deleterious [-4.37]^4^	Neutral [-2.37]^4^	Deleterious [-6.54]^4^

^1^The numbers in brackets represent the expected accuracy of the classification of each algorithm, as provided by PredictSNP.

^2^The numbers in brackets represent the disease probability according to SNPs&GO.

^3^The numbers in brackets represent the reliability of the classification of PMut.

^4^The numbers in brackets represent the score behind PROVEAN’s classification.

Despite affecting the same amino acid position, the R521C mutation exchanges a positively charged and basic amino acid for a non-charged and polar amino acid. On the other hand, variant R521H swaps a positively charged and basic amino acid for another positively charged and basic amino acid, which would possibly have less impact on protein structure and function. Particularly regarding that alteration in the protein net charge can affect its ability to interact with other molecules [42].

Moreover, two algorithms classified as neutral three mutations that are known to be disease-related, reinforcing the importance of using several prediction algorithms. Since these algorithms use different parameters to make predictions, using several algorithms can yield more reliable results, as previously shown by our group [43].

**Fig 1:**
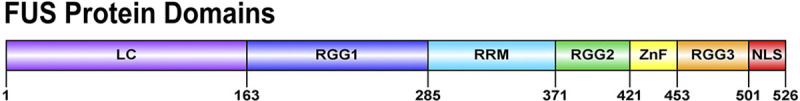
Graphic representation of the domains that compose the FUS protein. LC represents the low complexity, prion-like domain; RGG1 represents the first R-G-G pattern-rich domain; RRM represents the RNA-recognition motif; RGG2 represents the second R-G-G pattern-rich domain; ZnF represents the zinc-finger domain; RGG3 represents the third R-G-G pattern-rich domain, and NLS represents the nuclear localization signal.

Table 2 shows that none of the mutations were predicted to affect the aggregation tendency (TANGO) or amyloid propensity (WALTZ) of FUS. The human FUS protein is composed of seven domains (Fig 1), a prion-like region in its N-terminus that is rich in low-complexity (LC) amino acids, three regions with Arg-Gly-Gly motifs separating an RNA recognition motif, and a RanBP2-type zinc-finger domain [44], and a Pro-Tyr nuclear localization signal (PY-NLS) in its C-terminus [7]. Studies have shown that both the prion-like LC region [45] and the RGG motif regions [46] contribute to the self-association tendencies of the protein. These SNPeffect results corroborate this idea; since these mutations occur in the NLS, they would likely have no direct effect on aggregation tendency, according to the algorithm. Similarly, amyloid fibrils have been observed only in the LC region [47] and are also unlikely to be directly affected by the studied mutations.

**Table 2:** SNPeffect and FoldX effect predictions of FUS mutations.

	Protein Variants		
Predictive algorithms	R521C	R521H	P525L
TANGO	No effect [0.00]^1^	No effect [0.00]^1^	No effect [0.00]^1^
WALTZ	No effect [0.00]^2^	No effect [0.00]^2^	No effect [0.00]^2^
LIMBO	Increases [151.11]^3^	No effect [28.67]^3^	No effect [47.73]^3^
FoldX	Greatly reduces [-6.03 kcal/mol]^4^	Reduces [-3.9 kcal/mol]^4^	Reduces [-3.78 kcal/mol]^4^

^1^TANGO evaluates aggregation tendency, the number in brackets represents the numerical dTANGO value behind the algorithm’s classification.

^2^WALTZ evaluates amyloid propensity, the number in brackets represents the numerical dWALTZ value behind the algorithm’s classification.

^3^LIMBO evaluates chaperone binding tendency, the number in brackets represents the numerical dLIMBO value behind the algorithm’s classification.

^4^FoldX evaluates protein stability, the number in brackets represents the numerical ddG value behind the algorithm’s classification.

A hallmark of ALS is the formation of cytosolic aggregates. The composition of these aggregates depends mainly on the genetic mutation that is observed in the patient [48]. Aside from the mutated protein, other components can be found in these inclusions. For instance, misfolded wild-type TDP-43 is present in 97% of ALS cases [49]. Chaperones, specifically protein disulfide-isomerase (PDI), have also been observed as components in some FUS-positive inclusions [50], although the role of chaperones in ALS is still poorly understood. SNPeffect predicted an increase in chaperone binding tendency in mutation R521C, while R521H and P525L were unaffected.

### Protein frustration analysis

The results obtained from SNPeffect led us to investigate protein frustration in FUS since chaperones can mitigate protein frustration in the energy landscape of protein folding [51]. Protein frustration was best conceptualized by Ferreiro [52], where the author gives the example of a network of magnets that attract and repel each other according to their spins and the resulting energy state between pairs of magnets is termed frustration. Similarly, the amino acid residues that compose a protein have electrostatic charges and, in some cases, polarity, so the same concept can be applied.

**Fig 2:**
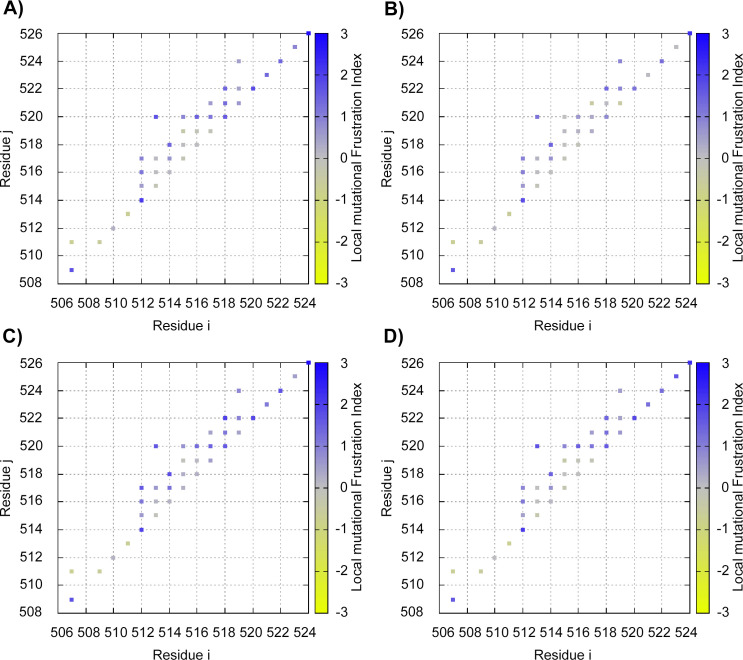
Mutational frustration analysis. Levels of mutational frustration by each residue-residue interaction. The colored scale represents the levels of frustration, in which blue represents minimal frustration, gray represents neutral frustration, and yellow represents high frustration. A) Wild-type FUS. B) R521C variant. C) R521H variant. D) P525L variant.

As shown in Fig 2, there is little difference in mutational frustration in the variants compared to the wild-type protein, except for variant R521C. In this case, there is a noticeable increase in frustration in interactions involving the mutated residue. This indicates that other amino acids would be more favorable in that position than the mutated cysteine (C). This change in frustration could also corroborate the SNPeffect results, as the elevated level of protein frustration could increase the need for chaperones to mitigate the frustration [51]. Interestingly, Farg *et al*. (2012) study indicated that protein disulfide-isomerases (PDIs) colocalizes with inclusions of R521C mutant FUS. PDI proteins are general protein chaperones and are also responsible for disulfide bond formation. High levels of PDIs were observed in ALS patients inside TDP-43, SOD1, and VAPB inclusions, playing a protective role against toxicity and misfolding. Thus, it is more likely that PDIs act as a chaperone refolding misfolded FUS than a disulfide bond inducer, given that this type of interaction has not yet been described before for FUS protein. However, it is possible that FUS protein, which contains four cysteine residues, may form non-native disulfide bonds, especially after R521C mutation providing an additional cysteine. Non-native bonds were already observed in mutated forms of SOD1 and TDP-43 proteins [50].

Interestingly, these results show that residue 521 interacts with residue 517, but also with residues 518, 519, and 523, which are binding residues for Karyopherin-β2 (Kapβ2), the importin that transports FUS, and other proteins, into the nucleus [20]. While it is unlikely that such electrostatic interactions would, by themselves, dramatically alter the structural stability of a protein [53], the significance of the affected residues could make them delicate to change. Especially since hydrophobicity is a hallmark of PY-NLSs [54] like the one present in FUS, and the amino acid cysteine is more hydropathic.

### Evolutionary conservation analysis

**Fig 3:**
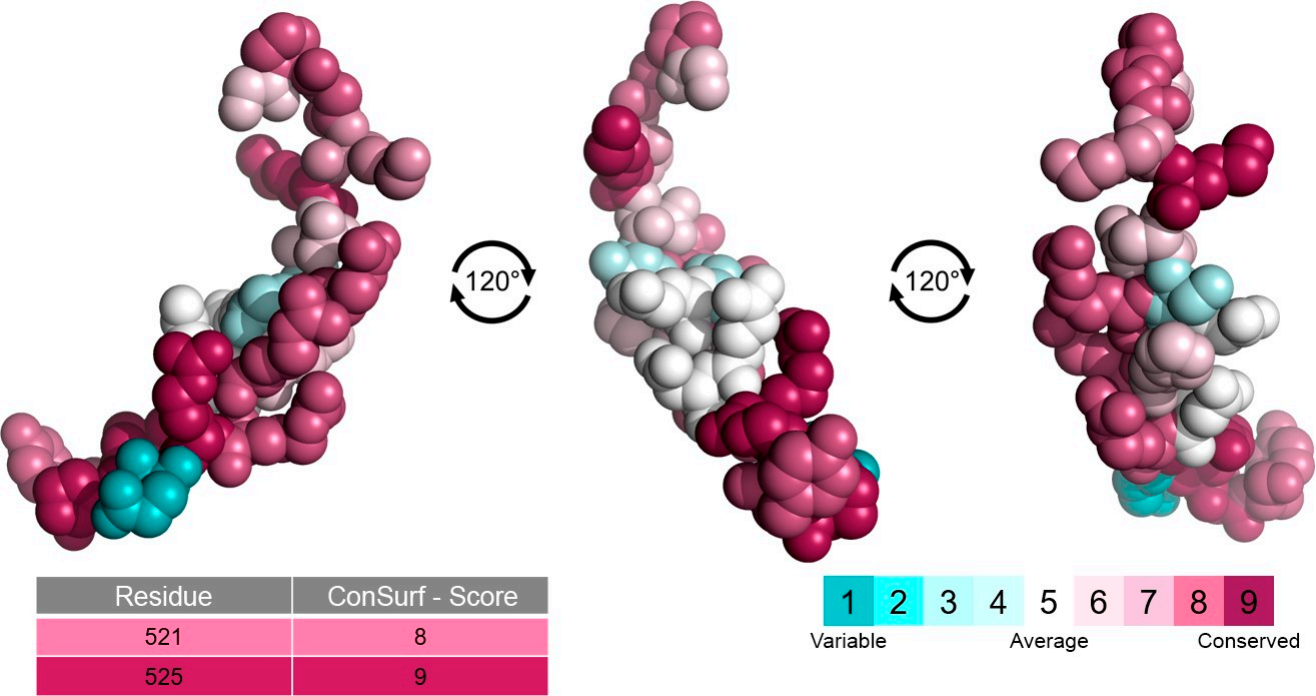
Evolutionary conservation analysis. Amino acid conservation analysis of wild-type FUS protein fragment (residue 507 to 526) represented in a space-filling model and colored according to conservation scores. Conservation scores of the residues where R521C, R521H, and P525L mutations occur are highlighted in the table. CONSURF color scheme representing conservation levels. Cyan represents more variable positions, while bordeaux represents more conserved positions.

CONSURF server analyses the evolutionary dynamics of amino acid residues between homologous sequences and gives each residue a score ranging from 1, more variable, to 9, more conserved [36]. Results showed that the amino acid residues in the NLS of the protein tend to be more conserved than variable, as shown in Fig 3. This is expected due to the relevance of the domain since areas that have an important role in protein function or structure tend to be more conserved [36]. Indeed, seven out of the ten residues in the NLS that interact with Kapβ2 were given conservations scores of seven or higher. Mutations in the NLS of FUS have been shown to weaken the affinity between FUS protein and the importin, and this loss of affinity causes the mislocalization of the protein to the cytosol, an effect that has been linked to ALS [55].

The amino acid residues where the studied mutations occur were also scored with high conservation levels by the algorithm. Residue 521 received a score of 8, while residue 525 received a score of 9. This corroborates that mutations occurring in more conserved sites tend to be more deleterious [56], seeing that variant P525L is frequently associated with a more aggressive disease phenotype [6]. Bulbar onset is more frequent in P525L than in other *FUS* mutations [6], which has been shown to have a smaller time frame between disease onset and death than spinal onset [57]. Additionally, the youngest case ever registered of ALS type 6 reported a carrier of the P525L *FUS* mutation with disease onset at merely 11 years of age [58], 28 years sooner than the median onset age for the condition [6].

### Molecular dynamics

During MD simulations, the trajectories of atoms and molecules are obtained by computational calculation of Newtonian equations of motion and application of different force fields [59]. Thusly, MDs can provide relevant information that would not be so easily discovered via experimental approaches [60], which tend to be much more costly. This type of information is also fundamental to improve efficiency and reduce the blindness of drug discovery [59].

RMSD is the average root-mean-square displacement of atoms between a reference structure and a dynamic structure, calculated at every frame of the simulation [37]. The reference structure is usually the first frame of the structure in the simulation, which was the case in this study. RMSD, thus, is a good measure of structural similarity throughout the simulation [61]. When a molecular system approaches equilibrium, the protein structure generally floats around an average stable conformation, and consequently, the RMSD values tend to form a plateau [62].

**Fig 4:**
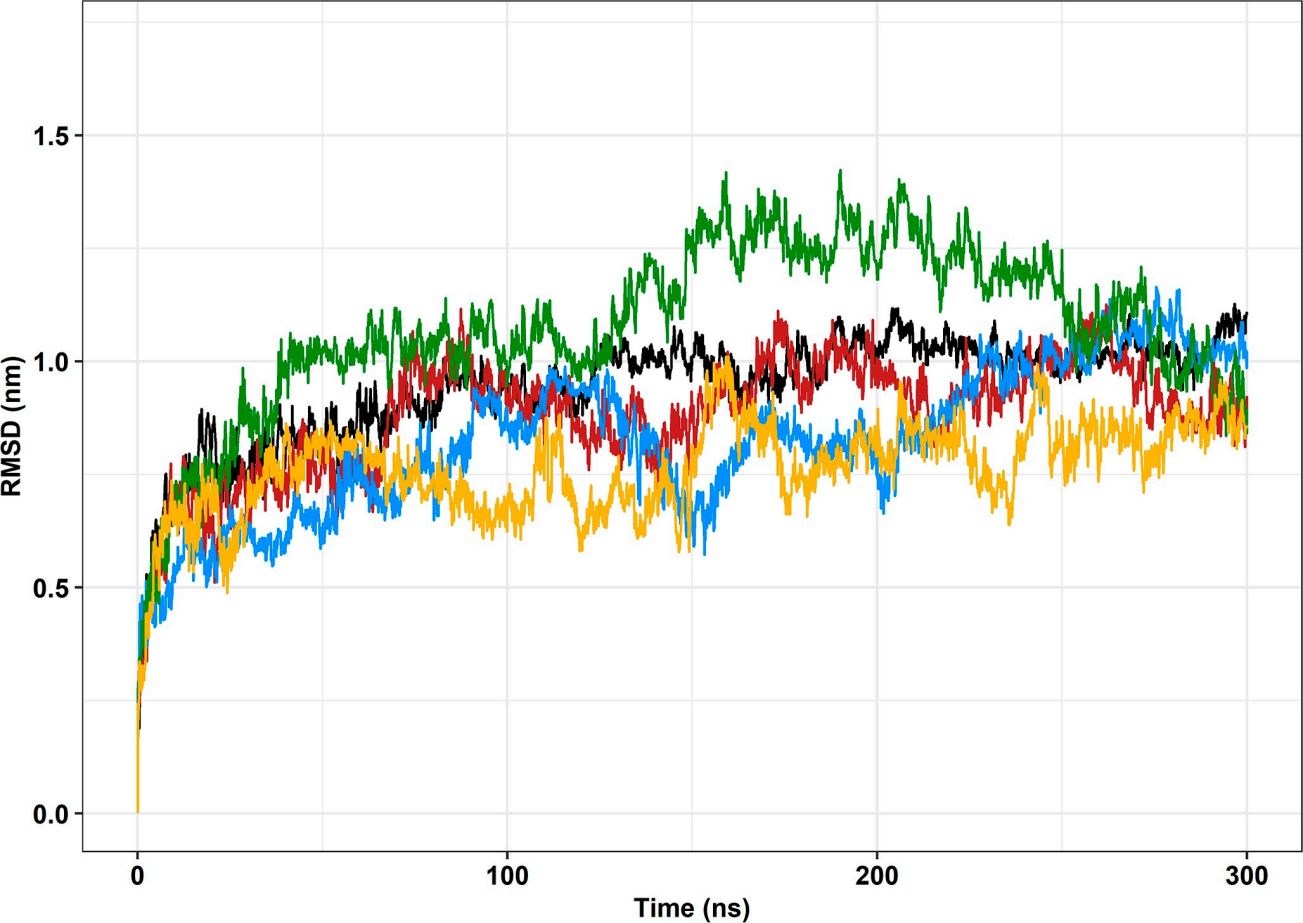
Root mean square deviation (RMSD) of the backbone atoms of the protein as a function of time. The wild-type FUS protein [PDB ID: 4FDD] is represented in black, variant R521C is represented in blue, variant R521H is represented in green, variant P525L is represented in red, and variant P525L-PDB [ID: 7CYL] is represented in yellow.

Wild-type FUS protein seemed to reach an equilibrium at around 125ns into the simulation when the RMSD value started fluctuating around 1nm, as shown in Fig 4. Variants P525L (induced *in silico*) and P525L-PDB [ID: 7CYL] were the only mutations to come close to an equilibrated state, which can be observed from the onwards of 160ns. These variants also presented similar behaviors throughout the simulations. R521C and R521H stand out by generally having lower and higher RMSD values, respectively, and seemingly did not achieve equilibrium during the simulations.

Root mean square fluctuation (RMSF) is similar to RMSD in that it measures the average displacement of atoms, but it does so relatively to individual residues throughout the simulation. Simply put, RMSF analyses flexibility differences for the amino acid residues [63].

**Fig 5:**
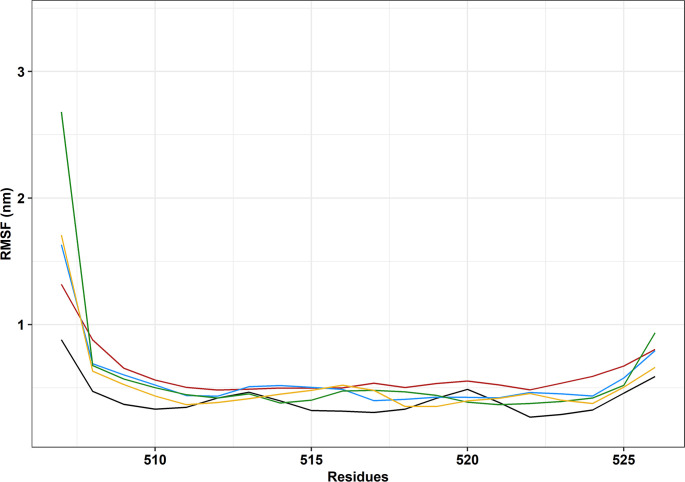
Root mean square fluctuation (RMSF) of the backbone atoms of the protein as a function of each amino acid residue. The wild-type FUS protein is represented in black, variant R521C is represented in blue, variant 521H is represented in green, variant P525L is represented in red, and variant P525L-PDB [ID: 7CYL] is represented in yellow.

Overall, variant P525L (induced *in silico*) was shown to have residues slightly more flexible than the other variants (Fig 5). Residue 507 was shown to be the most different between the variants and wild-type. While in the simulations of the P525L (induced *in silico*), P525L-PDB [ID: 7CYL], and R521C variants, residue 507 was approximately 33%, 60%, and 60% more flexible than the wild-type, respectively, this residue was nearly three times as flexible as the wild-type in the R521H simulation. Conversely, residue 513 showed the least variation of flexibility among all proteins, with all four receiving an RMSF value of approximately 0.5nm. Curiously, residue 507 is one of the sites that interact with Kapβ2 [20].

Aside from residue 507, none of the other amino acid residues that interact with Kapβ2 showed any distinct behavior other than that flexibility was generally higher in the variants than in the wild-type. Notably, alterations in flexibility can be very disruptive to proteins, putting at risk protein binding affinity and specificity [64], stability [65], and even misfolding [66], which is, by itself, linked to many diseases.

Radius of gyration (RG) is the mass-weighted root-mean-square distance of the atoms in a structure to their center of mass [20]. Thus, the radius of gyration can indicate the overall dimensions of a protein throughout an MD [63].

**Fig 6:**
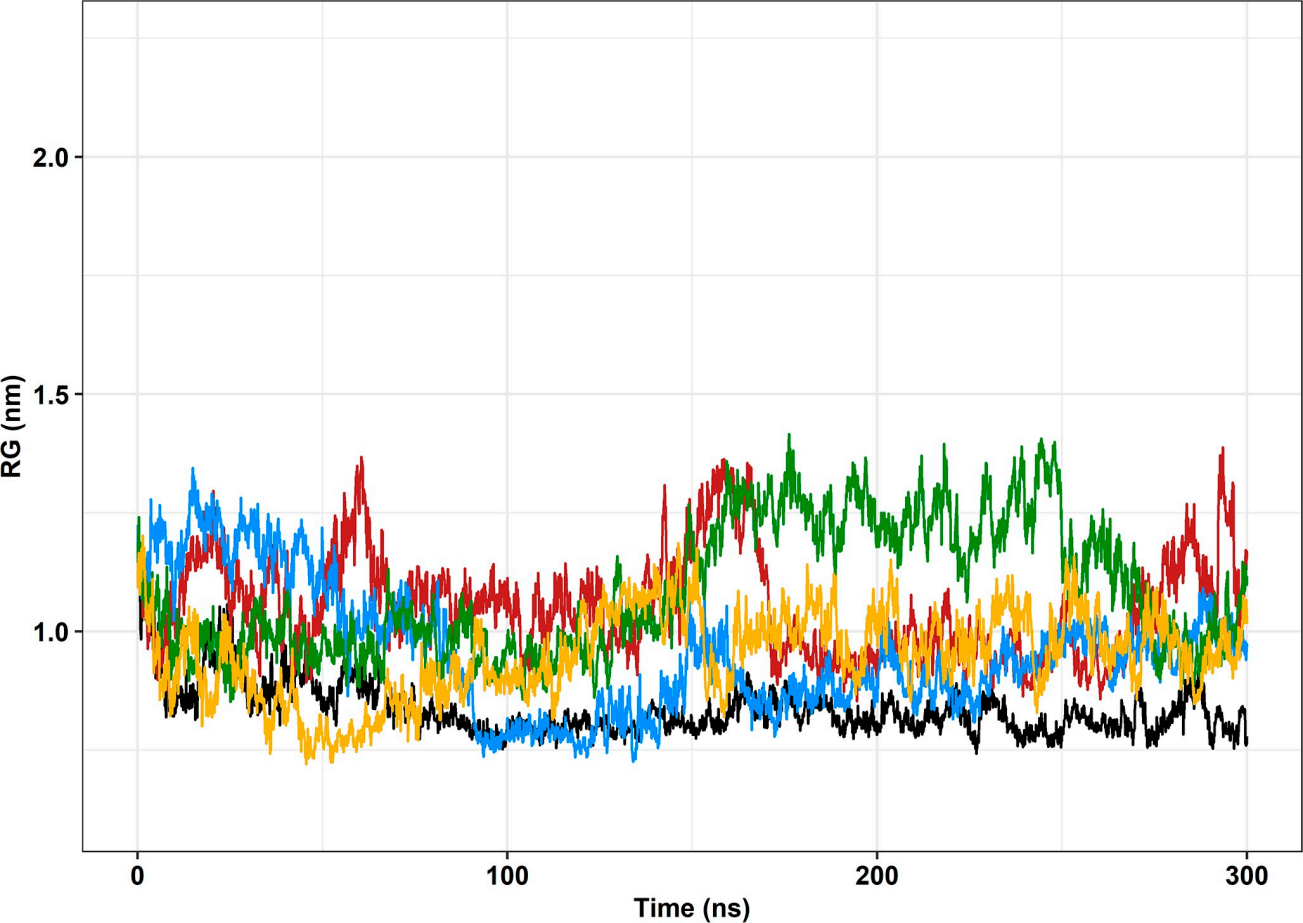
Radius of gyration (RG) of the backbone atoms of the protein as a function of time. The wild-type FUS protein is represented in black, variant R521C is represented in blue, variant 521H is represented in green, variant P525L is represented in red, and variant P525L-PDB [ID: 7CYL] is represented in yellow.

Wild-type FUS shows steady RG values, especially after 50ns, where the RG values fluctuate around 0.6nm until the end of the trajectory (Fig 6). Variant R521C presented steady RG values around 150ns, with values similar to those of the wild-type. Variant P525L-PDB [ID: 7CYL] also presented steady RG values around 150ns, however, with values slightly higher than those of the wild-type. Interestingly, variants R521H and P525L (induced *in silico*) have seemingly opposite behavior between 160ns and 290ns, but they do not present a steady behavior throughout the simulation. In general, R521H, P525L (induced *in silico*), and P525L-PDB [ID: 7CYL] presented less compactness than the wild-type fragment over time. Variant R521C showed similarities to the wild-type at certain points but was generally less compact, as well.

Solvent-accessible surface area (SASA) is the area of the protein that is exposed to solvent [67]. Thus, SASA can provide information about the ability of the protein to interact [63]. Interestingly, FUS tends to interact with other FUS proteins and self-assemble, forming the cytosolic inclusions that are believed to contribute to neurodegeneration in ALS [45].

**Fig 7:**
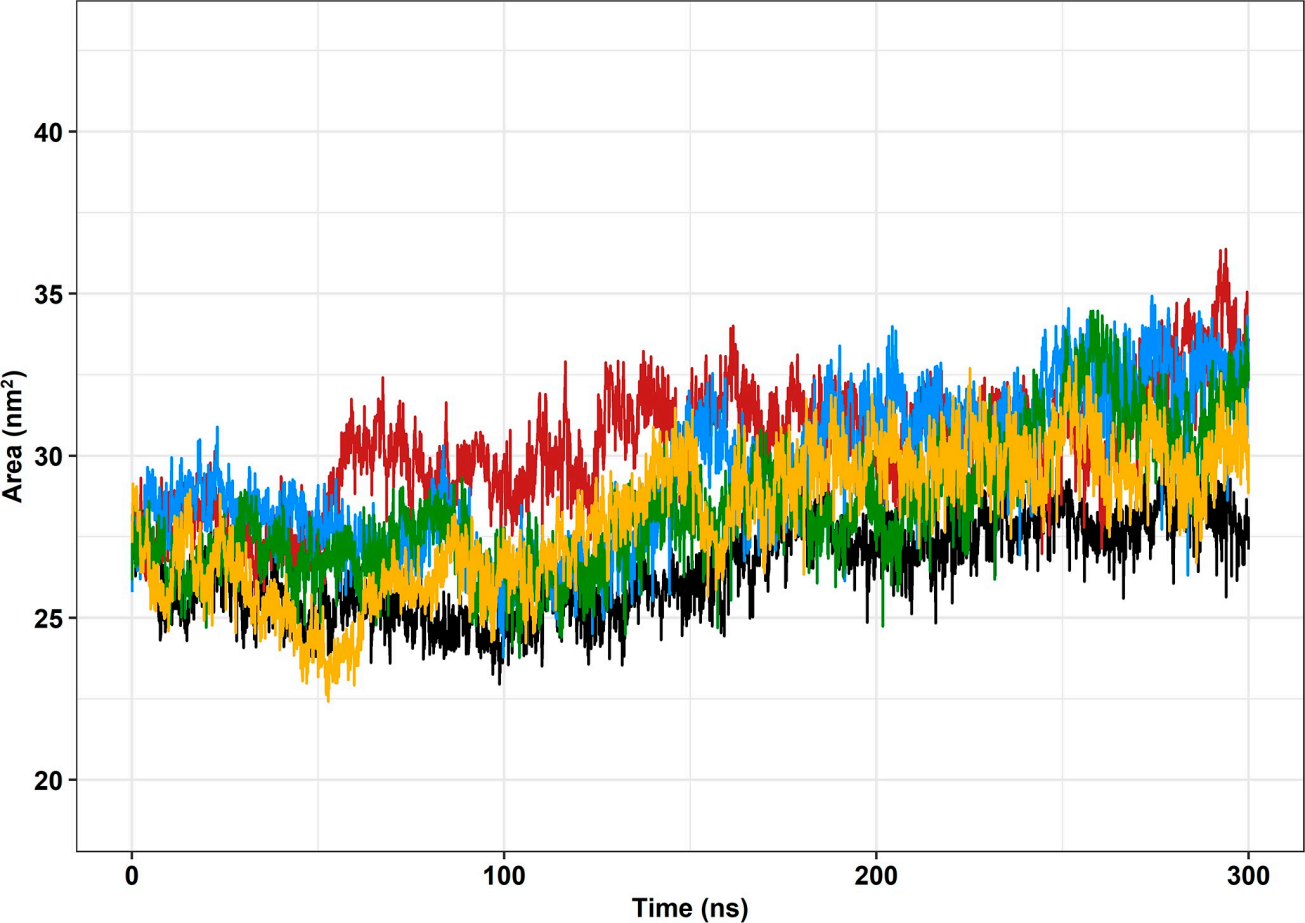
Solvent-accessible surface area (SASA) of the protein as a function of time. The wild-type FUS protein is represented in black, variant R521C is represented in blue, variant 521H is represented in green, variant P525L is represented in red, and variant P525L-PDB [ID: 7CYL] is represented in yellow.

Overall, all variant fragments were shown to have higher surface areas, which corroborates the RG analysis results that showed that the mutant fragments were generally less compact than the wild-type protein fragment, as seen in Fig 7. Moreover, similarly to the observed in the RMSD, and RG analyses, the SASA values for the wild-type were considerably steadier than those of the analyzed variants, which could further suggest that the variants R521C, R521H, and P525L could reduce protein stability [13]. This could also corroborate the results obtained from SNPeffect, which showed that all three mutations decrease protein stability.

The results obtained in this study showed that mutations R521C, R521H, and P525L could alter stability and flexibility for the FUS protein, which are factors essential to the function of a protein [65]. Flexibility, as previously mentioned, is an important factor in binding affinity and specificity [64]. Interestingly, the increase in flexibility observed in the studied mutations occur on several residues that interact with Kapβ2, the protein that carries transports FUS into the nucleus. Thusly, this increase in flexibility provides a possible link to the decrease in binding affinity between mutated FUS proteins and Kapβ2 [20]. By lowering the binding affinity between these two proteins, mutated FUS would be more likely to remain in the cytoplasm, as is observed in type 6 ALS patients [48].

MD simulations and functional predictions have been proven to be valuable for uncovering the effects of single mutations in proteins [68]. In addition to the methods presented in this paper, other powerful *in silico* approaches for the study of missense mutations are also available and worth mentioning. For example, the combination of classical MD outputs with advanced energy calculation methods (*i*.*e*. free energy perturbation, energy decomposition, MM/PBSA, and MM/GBSA) can provide relevant mechanistic information on the effects of mutations, particularly regarding protein interactions and structural stability [69–72]. Another widely used approach consists of combining MD simulations with unsupervised machine learning techniques such as clustering and principal component analysis. This approach allows researchers to assess representative conformations in proteins [69] and separate biologically relevant movements (*i*.*e*. opening, closing, and flexing) from small irrelevant local fluctuations, thus, providing detailed information on conformational dynamics [68].

## Conclusions

Through computational techniques, we were able to investigate how mutations R521C, R521H, and P525L affect the nuclear localization signal of FUS. Most of the functional prediction algorithms were capable of accurately predicting the three mutations as deleterious to the human organism. They also showed that the mutations decrease protein stability and, in the case of R521C, increase chaperone binding tendency. Relatedly, the protein frustration analysis showed an increase in frustration between the mutated R521C residue and the ones it interacts with. The evolutionary conservation analysis showed that the three mutations occur in highly conserved and probable important protein sites. The molecular dynamics results indicate that protein stability could be compromised in all three mutations. They also indicated that flexibility is elevated in most residues in all variants, most notably in the interaction site with the nuclear import protein of FUS. The analyzed mutations also affect the exposed surface area and protein compactness. These alterations in protein stability and flexibility could negatively impact the affinity between mutated FUS proteins and Kapβ2, leading to the formation of the FUS-positive cytoplasmic aggregates associated with type 6 ALS.

## Supporting information

S1 Dataset Minimal dataset. The tables contain the mean values used to create the molecular dynamics RMSD (Sheet 1), RMSF (Sheet 2), RG (Sheet 3), and SASA (Sheet 4) graphics, as well as the individual values obtained from each replicate. Sheet 5 contains the data output from ConSurf used in the evolutionary conservation analysis.
(XLSX)
